# A needle-mounted guide for improving needle visualization during ultrasound-guided needle insertion: a simulation study in novice operators

**DOI:** 10.1186/s40981-026-00873-5

**Published:** 2026-08-14

**Authors:** Yoshie Noji, Rieko Oishi, Takayuki Hasegawa, Shiori Tanaka, Satoki Inoue

**Affiliations:** Department of Anesthesiology, Fukushima Medical University, 1 Hikarigaoka, Fukushima City, Fukushima, 960-1295 Japan

**Keywords:** Ultrasound-guided procedures, Needle visualization, Simulation-based medical education

## Abstract

**Background:**

In ultrasound-guided needle insertion, continuous needle visualization is crucial for accurate needle placement, particularly for beginners. Novice operators may have difficulty maintaining the spatial relationship between the needle trajectory and the ultrasound imaging plane during freehand needle insertion. To address this educational challenge, we developed a detachable flat plate that can be attached to the needle shaft as a visual and tactile reference to assist alignment with the ultrasound imaging plane. The present study aimed to evaluate this plate's potential utility as an educational aid for novices performing both in-plane and out-of-plane techniques.

**Methods:**

This simulation-based randomized exploratory study included 32 practitioners who had no prior experience performing ultrasound-guided puncture. The participants were assigned to either the intervention group or the control group. In the intervention group, a custom-made flat plate was attached to the needle to assist alignment with the ultrasound imaging plane. After watching a brief video explaining how to do the procedure, the participants performed puncture tasks on a gelatin phantom at three probe positions—at the apex and at locations 45° to either side of the apex—using both in-plane and out-of-plane techniques. The primary endpoint was needle visualization during in-plane puncture at the right 45° position, assessed on a 5-point scale by a blinded evaluator. In addition, participants rated the ease of needle visualization using a 100-mm visual analogue scale.

**Results:**

Of the 39 individuals assessed for eligibility, 32 were included in the analysis (16 in the control group and 16 in the intervention group). All participants were right-handed except for one left-handed individual in the control group. The distributions of sex and participant background (medical students or nurses) were comparable between the two groups. No significant difference in objective needle-visualization scores was observed between the intervention and control groups (*p* = 0.0516), whereas self-assessed ease of needle visualization was significantly higher in the intervention group (*p* = 0.0157). In subgroup analysis within the intervention group, angled in-plane puncture showed a tendency toward higher objective and subjective scores, with no such tendency observed for the out-of-plane technique.

**Conclusions:**

Our needle-mounted guide improved subjective assessments of needle visualization and showed potential benefits during angled in-plane puncture. These findings suggest that the guide may help novice practitioners maintain alignment between the needle and the ultrasound imaging plane. Further evaluation in more homogeneous and standardized novice populations is required to assess the educational value and potential clinical applications of the device.

## Introduction

In ultrasound-guided needle insertion, continuous visualization of the needle is a critical factor for successful nerve blocks and vascular access procedures [1]. For example, to achieve continuous visualization during freehand needle insertion using the in-plane technique, the needle must remain aligned within the imaging plane generated by the center of the ultrasound probe [2, 3]. Even slight lateral deviation from this thin imaging plane can result in loss of needle visualization, making the procedure particularly challenging for beginners. Because ultrasound provides only a two-dimensional image of a three-dimensional operative field, novice operators often have difficulty understanding and maintaining the spatial relationship between the needle trajectory and the ultrasound beam [2, 3]. In addition, novice operators may experience similar difficulties in both the in-plane and out-of-plane techniques. In particular, in the out-of-plane technique, they may have difficulty reconstructing the three-dimensional spatial position of the needle tip based solely on point information in a two-dimensional ultrasound imaging plane.

As a result, beginners often rely on repeated trial-and-error and manual self-correction to acquire freehand ultrasound-guided needle insertion skills, resulting in a prolonged and inefficient learning process. To address this educational challenge, we sought to provide an additional visual and tactile reference on the needle itself rather than on the ultrasound probe. Specifically, we developed a detachable flat plate that can be mounted on the needle shaft.

The device is not intended to mechanically enforce precise needle alignment but rather to provide a broader visual and tactile reference surface than the needle shaft alone for the in-plane technique. By using the attached plate as a planar reference, operators may more easily recognize and correct needle orientation relative to the ultrasound imaging plane while preserving flexibility in needle angulation and directional adjustment during freehand needle advancement. We further hypothesized that this additional orientation cue might also facilitate spatial orientation during the out-of-plane technique by helping operators recognize the relationship between needle insertion direction and probe orientation.

Therefore, the aim of the presents study was to evaluate whether a simple needle-mounted orientation aid could improve needle visualization and the accuracy of needle alignment during simulated ultrasound-guided needle insertion performed by individuals with no prior experience in ultrasound-guided procedures for both the in-plane and out-of-plane techniques.

## Methods

### Study design and participants

This study was designed as a simulation-based exploratory study to evaluate the potential educational utility of a prototype needle-mounted guide for novice practitioners. Because the study did not involve patients, clinical procedures, or therapeutic interventions, it was not designed as a confirmatory clinical efficacy trial.

The study protocol was submitted to the UMIN Clinical Trials Registry (UMIN000058328) prior to participant enrollment; however, registration was not completed until after enrollment had begun. This simulation-based study did not involve patients or clinical interventions. The institutional ethics committee confirmed that formal ethics approval was not required. Verbal informed consent was obtained from all participants after an explanation of the study purpose and procedures.

Eligible participants included nurses working in operating rooms or intensive care units and medical students. Individuals with prior clinical experience in ultrasound-guided needle insertion or prior simulation-based training in ultrasound-guided needle insertion were excluded. Individuals with upper limb disabilities, visual impairments, or those who declined participation were also excluded. Participants were randomly assigned to either the intervention group or the control group.

### Construction of the add-on plate (Figs. 1, 2)

**Fig. 1:**
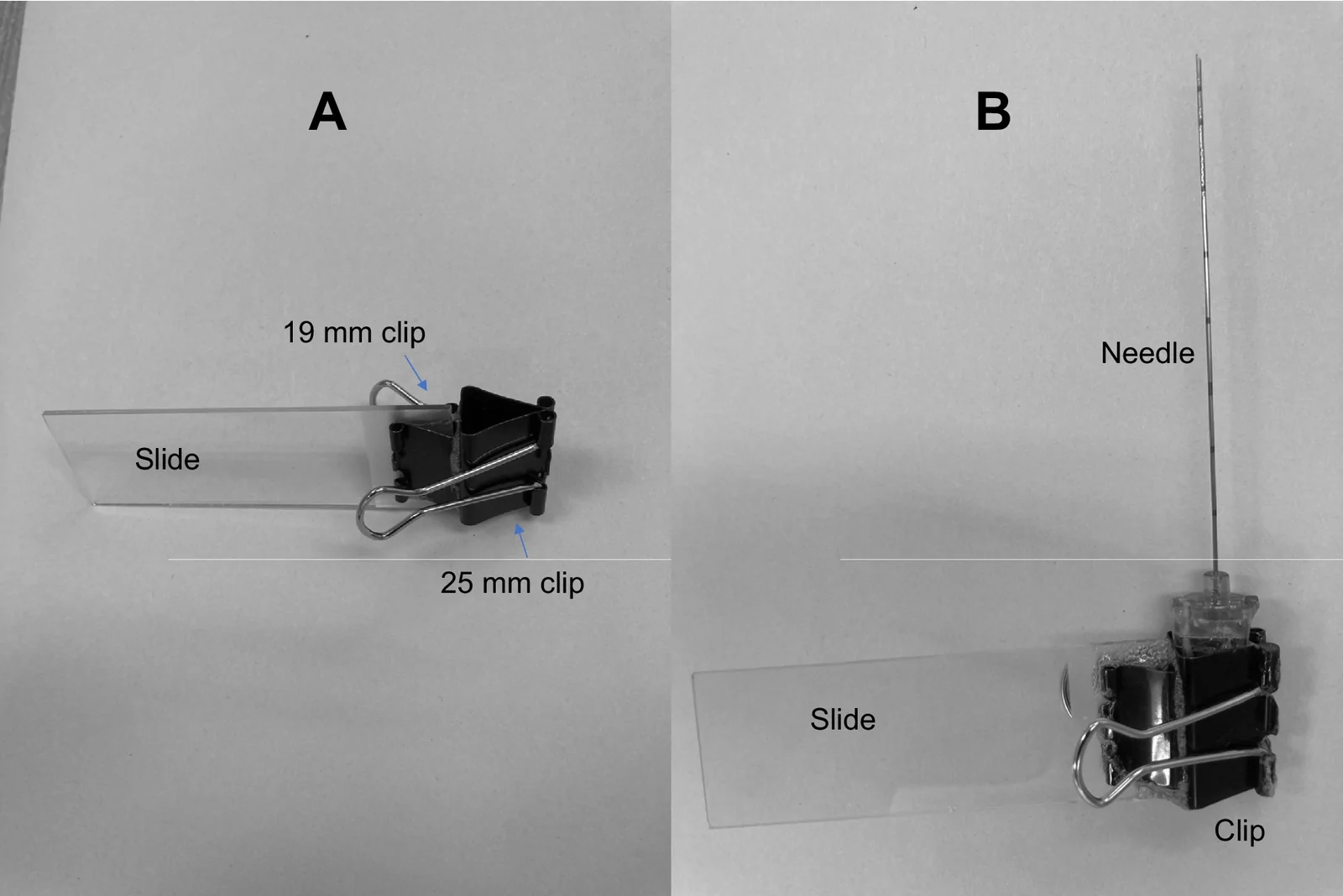
Construction of the add-on plate. Panel A shows the add-on plate constructed using 19 mm and 25 mm metal binder clips and a microscope slide. Panel B shows the add-on plate attached to the insertion needle by clamping the needle base with the 25 mm clip

**Fig. 2:**
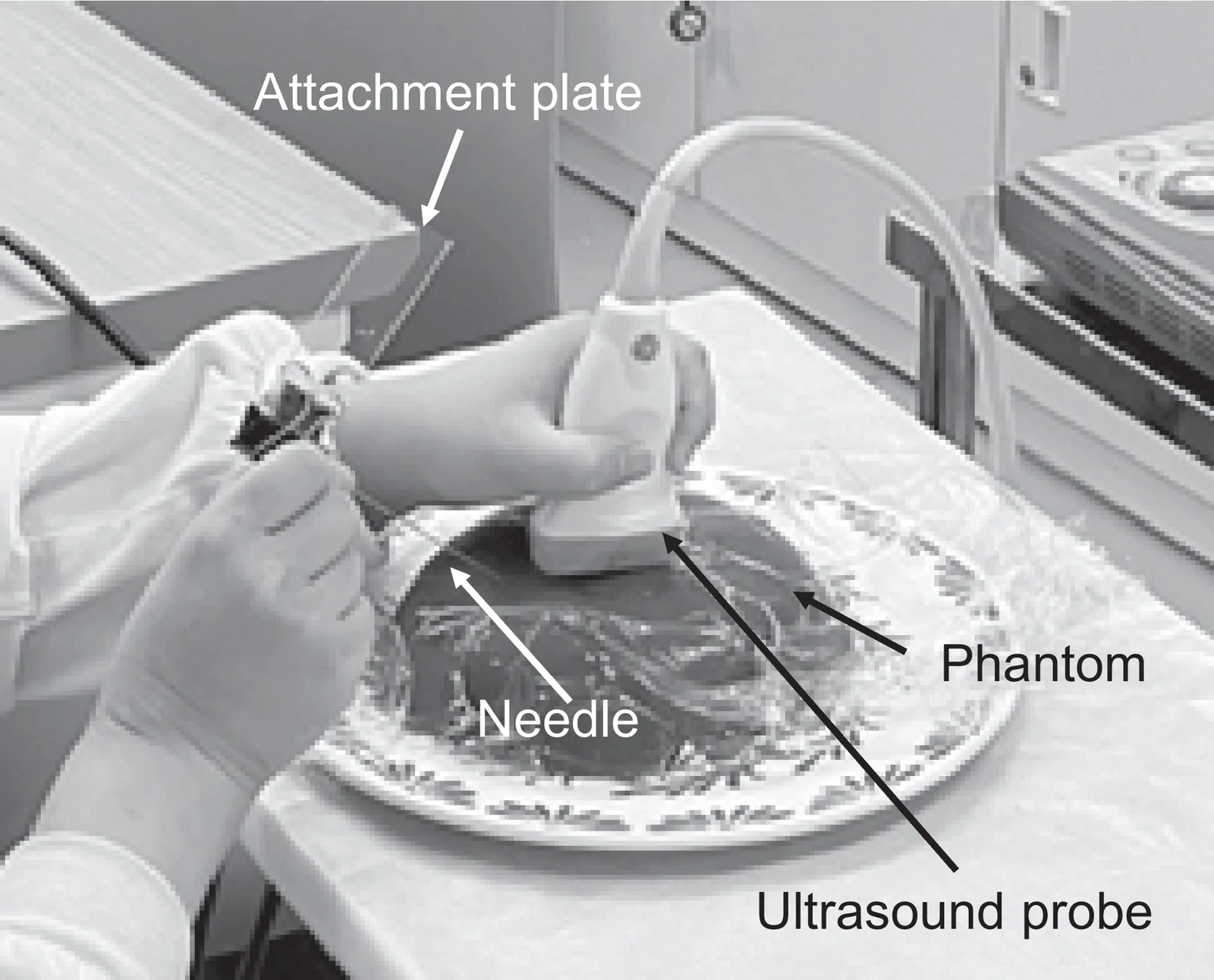
Experimental setup of the needle-mounted guide during ultrasound-guided puncture. Representative photograph showing the spatial relationship between the ultrasound probe, the gelatin phantom, and the needle equipped with the attached plate during puncture. The operator’s hand position and device orientation relative to the ultrasound imaging plane are shown, confirming that no physical interference occurred between the plate and the ultrasound probe during needle advancement

The prototype add-on plate was constructed using commercially available 19 mm and 25 mm metal binder clips and a microscope slide. First, the handles were removed from the 19 mm clip, and the microscope slide was attached to the clip with adhesive. The backs of the 19 mm and 25 mm clips were then bonded together so that the slide was positioned approximately parallel to the needle shaft. The completed assembly was mounted by clamping the proximal portion of the insertion needle with the 25 mm clip.

The device was designed as a simple prototype intended to provide a visual and tactile reference for needle alignment during simulation-based training rather than to function as a precision-engineered guidance instrument.

### Ultrasound settings

Ultrasound imaging was performed using a 12L-RS linear probe (GE HealthCare Japan) connected to a Vivid i ultrasound system, with the frequency set to 12 MHz and an imaging width of 39 mm. The ultrasound settings were standardized for all participants and included an imaging depth of 40 mm, a single focal zone, a gain of 74 dB, and a frame rate of 42 Hz. The imaging depth was standardized at 40 mm for all participants.

An 80 mm 22 G needle (Ultraplex 360, B. Braun Aesculap, Tokyo, Japan) was used for all procedures. A standard echogenic needle commonly used in ultrasound-guided procedures was selected because its length and echogenic characteristics were suitable for standardized simulation of both in-plane and out-of-plane needle insertion tasks.

### Pre-training for participants

Participants received approximately 4 min of standardized video-based instruction developed by the investigators. The instructional video did not include use of the needle-mounted plate device. No hands-on training was provided. The educational content included the following key concepts: (1) B-mode ultrasound generates images from a thin, two-dimensional imaging plane; (2) for the in-plane technique, the needle trajectory must be aligned within this imaging plane (co-planar alignment) to ensure continuous visualization of the entire needle shaft; and (3) for the out-of-plane technique, the needle should be advanced toward the center of the ultrasound beam and maintained perpendicular to the imaging plane, allowing the needle tip to be visualized as a focal hyperechoic point.

Participants assigned to the intervention group additionally viewed a supplementary instructional video (approximately 1 min) demonstrating the use of the attached plate. This video explained how the device was intended to help maintain co-planar alignment during the in-plane technique and facilitate a consistent perpendicular needle trajectory relative to the ultrasound imaging plane in the out-of-plane technique.

### Ultrasound phantom preparation

A tissue-mimicking phantom was fabricated using a mixture of 750 mL of water, 45 g of gelatin, 15 g of mannan, and 22.5 g of psyllium. The gelatin and mannan were dissolved in heated water, followed by the addition of psyllium and food coloring. This solution was cast into a 20-cm-diameter metal mold and refrigerated for at least 8 h to solidify. The resulting phantom exhibited a flat base, providing stable support and a curved, elastic upper surface. This geometry was intended to render the realistic variability in probe positioning during ultrasound-guided procedures.

The phantom was placed on a table set at a height of 75 cm to simulate real-world conditions. Because the phantom gradually deteriorates with repeated use, it was replaced after every six to seven uses and discarded at the end of each day to maintain consistent experimental conditions across participants.

### In-plane technique

The in-plane technique was evaluated under three standardized probe positions: (1) a neutral position in which the probe was placed directly above the topmost point of the spherical phantom, which served as the reference point for probe positioning, and (2) two oblique positions at approximately 45° to the left and right relative to the operator. These oblique positions were introduced to create different levels of difficulty in coordinating needle manipulation with probe orientation during ultrasound-guided needle advancement. The operator’s line of sight and hand position were kept as consistent as possible across conditions to minimize variability in hand–eye coordination, although maintaining this consistency was inherently more difficult in the oblique positions.

The spherical gelatin phantom provided a curved surface that introduced variability in probe stability during ultrasound-guided needle insertion. The topmost point of the phantom was used as the reference point for positioning the ultrasound probe in the neutral condition. The ultrasound probe was oriented such that its long axis was directed toward the predefined target point and approximately aligned with the operator’s forward-facing direction. All experiments were conducted in a seated position. The ultrasound monitor and the phantom were placed on separate tables, each approximately 75 cm in height, such that the monitor and the puncture site were positioned at approximately the same vertical level. Participants performed the task while seated on a standard chair approximately 40 cm in height, and the chair height was not adjusted between participants. The positions of the chair, phantom, and ultrasound monitor were standardized using floor markings and were returned to the predefined positions whenever displacement occurred. Although operators naturally shifted their gaze between the phantom and the ultrasound monitor during the task, the relative positions of these components were kept constant throughout the study to minimize variability in hand–eye coordination and viewing conditions.

The needle entry point was fixed approximately 20 mm lateral to the ultrasound imaging field. Rather than targeting a specific structure within the phantom, participants were instructed to advance the needle across the ultrasound image toward a virtual target located approximately 30 mm deep near the far lateral edge of the ultrasound imaging field. The target depth was identified using the ultrasound depth scale (imaging depth: 40 mm), thereby providing an opportunity for continuous needle visualization throughout the task. In the plate-assisted condition, participants were instructed to use the attached plate as a visual and tactile reference to maintain approximate alignment between the needle and the ultrasound imaging plane during advancement. Each trial lasted 10 s, beginning after the examiner confirmed correct initial positioning of the probe and needle and verbally instructed the participant to begin the task. The fixed time limit was introduced to standardize task duration across participants and reduce variability associated with unrestricted exploration time. No feedback was provided during the task.

### Out-of-plane technique

The probe was placed at the same three physical positions used in the in-plane task: at the topmost point of the phantom's surface and at approximately 45° to the left and right of the operator. The probe was oriented with its short axis facing the operator, with the probe orientation rotated by 90° relative to the in-plane task. The virtual target location was defined as a screen-based coordinate aligned with the center of the ultrasound imaging field.

The ultrasound screen was positioned directly in front of the operator to maintain a consistent line of sight during the procedure. Participants inserted the needle perpendicular to the probe face from an insertion point approximately 30 mm from the probe edge. This arrangement was selected to provide a standardized spatial orientation task for novice participants. Participants adjusted the needle trajectory to bring the needle into view at a depth of approximately 20 mm along the central vertical axis of the ultrasound imaging field, as indicated by the depth scale. No discrete target was embedded within the phantom. In the plate-assisted condition, participants were instructed to use the attached plate as a visual reference to maintain an approximately perpendicular relationship between the needle trajectory and the probe orientation during advancement. Trial duration, task initiation procedures, and feedback conditions were identical to those used for the in-plane technique.

For both the in-plane and out-of-plane tasks, participants were not instructed to use a specific hand for holding either the ultrasound probe or the needle, and hand assignment was not recorded.

### Evaluation methods

#### Self-assessment

Participants self-assessed the ease of needle visualization under ultrasound guidance using a 100-mm visual analogue scale (VAS), where 0 represented extremely difficult and 100 represented extremely easy.

#### Objective evaluation

The extent to which the needle could be visualized on ultrasound images within the allotted time was evaluated for both the in-plane and out-of-plane techniques. The extent to which the needle could be visualized on ultrasound images within the allotted time was evaluated for both the in-plane and out-of-plane techniques. An objective evaluation was conducted using video recordings of the ultrasound monitor display. A single independent evaluator, blinded to the type of needle condition, assessed needle visualization using a predefined 5-point ordinal scale based on the recorded videos. Video recordings were obtained using a smartphone mounted on a fixed selfie stick positioned approximately 50 cm behind the participant. The smartphone field of view was adjusted so that only the ultrasound monitor screen filled the recording frame, with the operator’s body, hands, and any procedural devices excluded to ensure assessor blinding. The ultrasound display settings were standardized across all participants, including depth and zoom levels, to ensure consistent image scaling. Because direct digital image capture from the ultrasound system was not available, external video recording was used for outcome assessment.

##### In-plane technique

The needle tip was identified according to standard clinical criteria for ultrasound-guided needle visualization.

The 5-point evaluation scale was as follows:

1. No needle was visible.
2. Only a small part of the needle was visible.
3. Half of the needle was visible, but the tip near the predefined reference point was not visible.
4. More than half of the needle was visible, including the needle tip near the predefined reference point.
5. The entire needle was visible, including the needle tip at the predefined reference point.

##### Out-of-plane technique

Because the recordings were analyzed frame-by-frame, the frame in which the advancing needle tip was first clearly visualized within the ultrasound image could be identified. This allowed determination of both the lateral position and depth of the needle tip at the time of visualization.

Spatial evaluation was conducted using the on-screen ultrasound depth scale and the central image axis as the reference. Measurements were obtained from the frame in which the needle tip was first clearly visualized, and these measurements were used to categorize lateral deviation into the predefined scoring ranges. Horizontal displacement was estimated using the vertical depth scale as a reference, as no lateral scale is displayed on standard ultrasound images. The 5-point scale for the out-of-plane technique was as follows:

1. Deviation > 10 mm from the midline
2. 5 mm < Deviation ≤ 10 mm
3. 2.5 mm < Deviation ≤ 5 mm
4. Deviation ≤ 2.5 mm (needle tip depth outside the range of 10–30 mm)
5. Deviation ≤ 2.5 mm and needle tip depth between 10 and 30 mm

Prior to formal assessment, the evaluator was trained using representative reference images illustrating each score level. The evaluator remained blinded to group allocation throughout the assessment process. Lateral deviation and needle tip depth were categorized into predefined clinically relevant ranges rather than recorded as continuous numerical variables.

### Primary endpoint

The primary endpoint was the objective performance score during the in-plane task performed under a right 45° probe angulation, assessed using a 5-point ordinal scale of needle visualization. This condition was selected because needle visualization can be assessed more straightforwardly in the in-plane technique than in the out-of-plane technique and because the in-plane technique provides stable and reproducible imaging conditions for objective evaluation.

In addition to the flat surface condition at the apex of the phantom, performance was also evaluated on the spherical portion of the phantom. The spherical surface combined with right 45° probe angulation was included to simulate a less stable imaging environment, in which maintaining alignment between the needle and the ultrasound imaging plane is expected to be more difficult for novice operators. This configuration was selected based on preliminary pilot testing to reflect a more challenging in-plane condition.

### Sample size

For sample size estimation, we assumed a clinically meaningful difference of 2 points in the primary endpoint (5-point objective evaluation score during the in-plane task performed under a right 45° probe angulation). Because no prior data were available for this specific device, the assumed effect size (defined as the absolute between-group difference in the primary endpoint) was based on pilot observations in novice participants. A standard deviation of 2 points was assumed.

Using a two-sided α level of 0.05 and power of 80%, the required sample size was calculated as 16 participants per group. A standard normal approximation was used due to the absence of prior distributional data. This study was designed as a pilot feasibility study to inform future confirmatory trials.

### Statistical analysis

Participants’ dominant hand, sex, and professional status were reported as frequencies, while age was expressed as the mean and standard deviation. Self-assessed VAS was expressed as the median and interquartile range because of the small sample size and the potential for non-normal distribution. The objective evaluation of needle visualization was also summarized as the median and interquartile range because it was based on an ordinal 5-point scale. The primary analysis compared the objective needle visualization score between the intervention and control groups during the in-plane technique at the right 45° probe position on the spherical phantom using the Mann–Whitney U test. Secondary analyses included comparisons of self-assessed VAS scores under the same condition and objective and subjective outcomes during the out-of-plane technique using the same statistical method. Although inter- or intra-group comparisons were not performed for repeated measures of VAS and needle insertion assessments to avoid analytical complexity and a potential reduction in statistical power, the descriptive values (not *p*-values) are presented for reference. A *p*-value of < 0.05 was considered statistically significant.

## Results

**Fig. 3:**
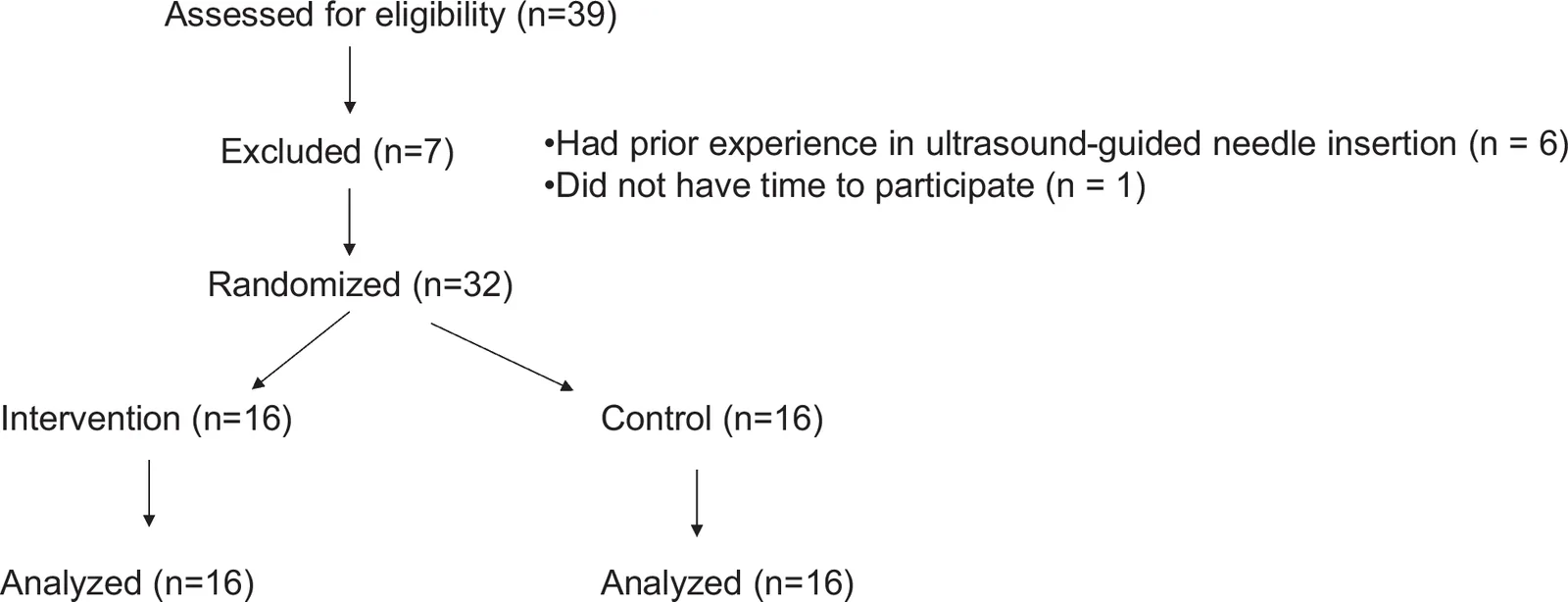
Participant flow diagram of the study. Of 39 individuals assessed for eligibility, seven were excluded (6 due to prior experience with ultrasound-guided needle insertion and one due to refusal). The remaining 32 participants were randomly assigned to the control and intervention groups and included in the final analysis

A total of 39 individuals were assessed for eligibility. Six individuals were excluded due to prior experience with ultrasound-guided procedures, and one declined participation due to time constraints. Thirty-two participants were randomized equally to the control and intervention groups (*n* = 16 each) and included in the final analysis (Fig. 3). The control group comprised seven medical students and nine nurses, whereas the intervention group comprised four medical students and 12 nurses.

There were nine men and seven women in the control group and seven men and nine women in the intervention group. Only one participant in the control group was left-handed; all other participants were right-handed.

The mean (standard deviation) age was 28 (9) years in the control group and 31 (9) years in the intervention group.

**Fig. 4:**
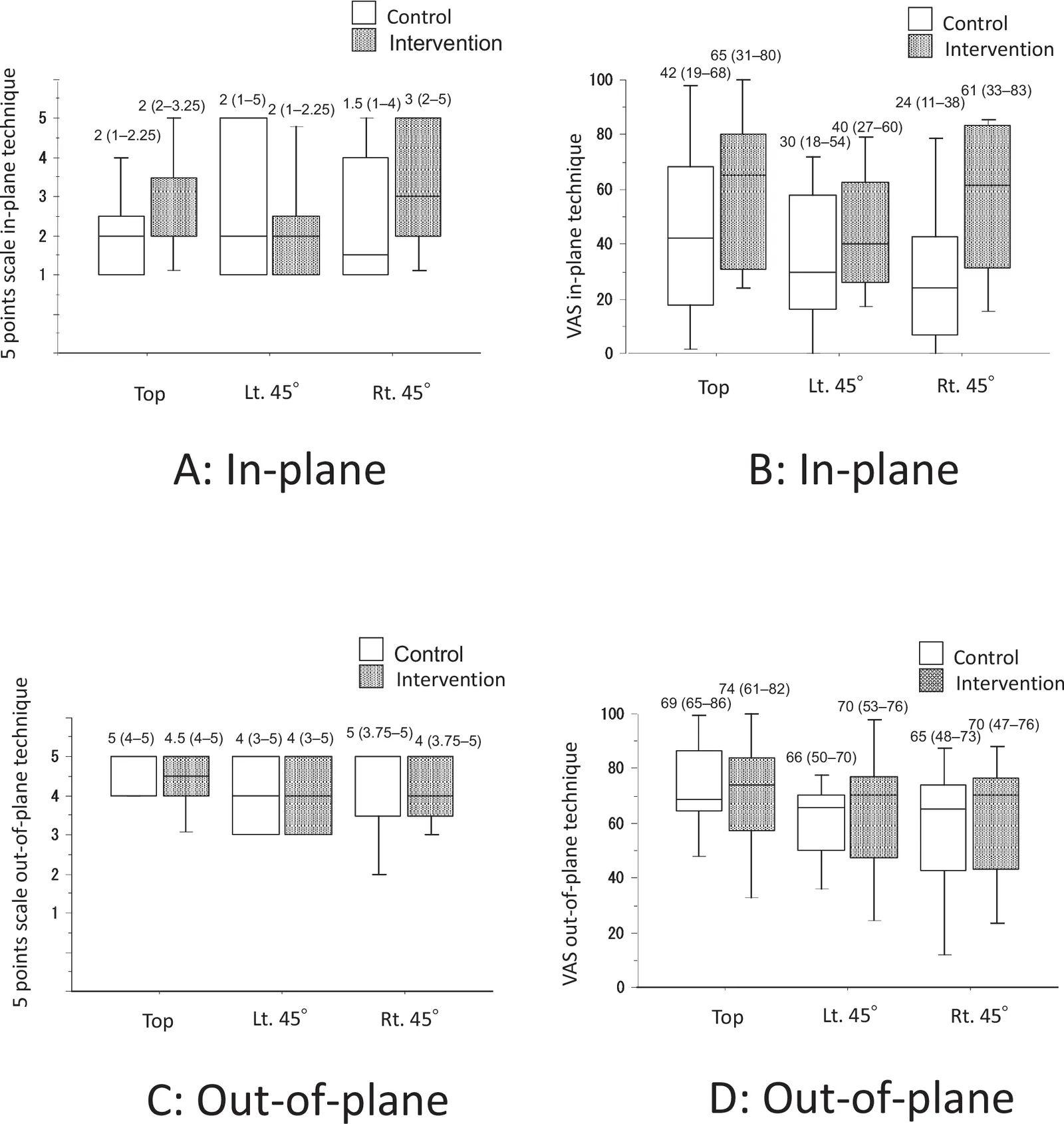
Objective and self-assessed needle visualization across probe orientations. Panels A and B show objective needle visualization (VAS) and self-assessed ease of needle visualization (5-point scale) using the in-plane technique in both groups. Panels C and D show corresponding results using the out-of-plane technique. Each box represents the interquartile range (IQR), with the median indicated by a horizontal line; whiskers extend to the minimum and maximum values within 1.5 × IQR. The numerical values above each box indicate the median and IQR. VAS, visual analog scale

Table 1 summarizes the primary endpoint condition (in-plane technique under right 45° probe angulation), whereas Fig. 4 presents the distribution of outcomes across all experimental conditions for exploratory comparison.

**Table 1:** Objective and subjective needle visualization outcomes for the primary endpoint (in-plane technique under right 45° probe angulation)

	Control	Intervention	*p* value
In-plane technique			
Objective assessment (5-point scale)	1.5 (1–4)	3 (2–5)	*p* = 0.052
Self-assessment (VAS)	24 (11–38)	61 (33–83)	*p* = 0.016
Out-of-plane technique			
Objective assessment (5-point scale)	5 (3.75–5)	4 (3.75–5)	*p* = 0.476
Self-assessment (VAS)	65 (48–73)	70 (47–76)	*p* = 0.585

Values are presented as median (interquartile range). Each participant contributed one measurement per condition. The primary outcome represents performance during the in-plane task under the right 45° probe angulation. The left and right 45° positions were not pooled for analysis. VAS indicates visual analog scale

Table 1 presents the results of the primary analysis and related outcome measures. The intervention group showed no significant difference in objective needle visualization during the in-plane technique compared with the control group. However, self-assessed needle visualization scores were significantly higher in the intervention group (*p* < 0.05). Regarding the out-of-plane technique, no significant differences were observed between groups in either objective or self-assessed needle visualization scores. The needle remained within the ultrasound imaging field during the assessment, allowing the needle tip or shaft to be visualized on the screen.

Figure 4 summarizes objective and self-assessed needle-visualization scores for both the in-plane and out-of-plane techniques in the control and intervention groups. In the in-plane technique, the intervention group tended to achieve higher objective and subjective scores at the 45° positions. Conversely, such a tendency was not observed with the out-of-plane technique.

## Discussion

This study investigated the educational utility of a needle-mounted guide for novice practitioners learning ultrasound-guided needle insertion. Although no statistically significant differences were observed in the objective needle-visualization score between groups, participants reported significantly greater ease of needle visualization when using the guide. The magnitude of the between-group difference suggested a potential educational benefit, although this should be interpreted cautiously given the exploratory nature of the study. Therefore, the device may serve as a simple educational aid for novice practitioners learning freehand ultrasound-guided needle alignment.

Ultrasound-guided needle insertion using the in-plane approach is technically challenging because continuous visualization of the needle requires accurate alignment within the ultrasound imaging plane [4, 5]. Although operators may initially achieve appropriate alignment, maintaining it throughout needle advancement can be difficult, particularly for novice practitioners. The present prototype was designed to provide visual and tactile cues that facilitate recognition and maintenance of this alignment during freehand needle insertion.

In contrast, the needle-mounted guide did not significantly affect either objective or subjective needle-visualization scores during the out-of-plane technique. Previous studies have suggested that the out-of-plane approach may be relatively straightforward for inexperienced operators, although interpretation of the echogenic needle image remains challenging [6, 7]. Because the participants in this study were novice practitioners with no prior experience in ultrasound-guided needle insertion, they may have had difficulty interpreting the spatial position of the needle during the task. In addition, the objective assessment in this study focused on overall needle image visibility rather than precise identification of the needle tip, which may have limited the ability to detect between-group differences.

As described above, the primary outcome did not demonstrate a statistically significant difference between groups. However, a non-significant trend in the between-group difference was observed, suggesting a potential but modest educational effect. Considerable variability was observed among participants, which is expected given the inclusion of true novices with no prior experience in ultrasound-guided needle insertion. The study was designed as an exploratory simulation-based educational investigation, and participants were intentionally limited to novices to reduce heterogeneity related to clinical background and procedural familiarity. Nevertheless, the lack of prior exposure to ultrasound-guided techniques may have contributed to variability in both performance and self-assessment. Future studies may benefit from incorporating a brief standardized introductory training session prior to evaluation, which could help establish a minimal level of procedural understanding while preserving the learning characteristics of novice participants. Further research is needed to determine the optimal training structure and target population for evaluating educational support devices of this type.

In the present out-of-plane task, participants were primarily required to advance the needle toward the center of the ultrasound image. Because only a cross-sectional echogenic view of the needle was available, participants could adjust needle position during advancement based on the observed image. As a result, the task did not require precise recognition of the full needle trajectory or continuous spatial tracking of the needle outside the imaging plane. Therefore, the current experimental design may have limited the ability to detect potential educational benefits of the needle-mounted guide during the out-of-plane technique. A task requiring more precise initial spatial alignment without repeated positional correction might be better suited to evaluate whether the guide facilitates understanding in novice practitioners of the relationship between needle direction and probe orientation during out-of-plane needle insertion.

The mechanism underlying the observed improvement remains speculative. The attached plate was not intended to mechanically enforce exact needle alignment; rather, it may have functioned as a broader visual and tactile reference surface that facilitated perception of needle orientation relative to the ultrasound imaging plane. Compared with the needle shaft alone, the planar surface may have enhanced spatial awareness and aided trajectory correction during freehand needle advancement. Accordingly, the potential benefit of the device may depend more on facilitating perception of orientation than on achieving precise geometric alignment. Further studies are needed to clarify the relative contributions of visual guidance, tactile feedback, and mechanical stabilization to the observed effects.

Although no statistically significant differences were observed in the primary outcomes, the findings suggest that the proposed concept may have potential as a supportive educational aid for novice operators in simulated environments. However, these findings should be interpreted in light of the study's exploratory nature, as it was intended as a preliminary exploration of an educational concept rather than a definitive efficacy trial.

This study has several limitations. First, the simulation task used a virtual target defined on the ultrasound image rather than anatomically defined structures such as simulated nerves or blood vessels. More clinically recognizable targets might have provided clearer spatial objectives for novice participants and could have influenced task performance.

Second, variability in inherent psychomotor and spatial abilities among participants may have influenced the results. Although a paired or crossover design might reduce inter-individual variability, such designs may also be affected by learning effects during repeated ultrasound-guided needle insertion tasks. Future studies therefore require careful selection of study populations and experimental designs when evaluating educational support devices.

Third, although the elasticity of the gelatin phantom generally allowed stable probe contact despite the curved surface geometry, probe stability was not objectively assessed in this study. Therefore, the potential influence of probe handling on task performance cannot be completely excluded. Future studies incorporating objective measures of probe stability or comparing different phantom geometries may help clarify the contribution of this factor.

Fourth, the simulation environment used in this study was intentionally designed to be challenging and did not directly replicate routine clinical ultrasound-guided procedures. The combination of probe angulation and curved phantom geometry may limit the generalizability of the findings to clinical practice. Further studies in more clinically representative training environments are needed to determine whether the observed benefits can be translated to real-world ultrasound-guided procedures.

Fifth, ultrasound images were evaluated using external video recordings of the ultrasound monitor rather than direct digital export from the ultrasound system. Although recording conditions, monitor settings, and camera positioning were standardized across all experiments, this approach may have reduced image fidelity and introduced environmental factors that could affect needle visualization. Future studies employing direct digital image capture may provide more precise assessment of needle visibility. In addition, distance categorization was based on the displayed ultrasound scale rather than dedicated digital measurement software.

Another limitation is that participants were not instructed to use a standardized hand assignment for holding the ultrasound probe and needle, and hand assignment was not recorded. Because ergonomic difficulty may differ depending on hand assignment and probe orientation, particularly for left-handed operators, this factor could have influenced task performance. Future studies should standardize or record hand assignment to evaluate its potential effect.

Finally, the needle-mounted guide used in this study was a custom-made exploratory prototype constructed from readily available materials. Accordingly, no formal calibration or quantitative verification of needle-guide alignment was performed, and exact parallelism between the needle and the attached plate could not be assured. Minor deviations may have occurred due to assembly variability, needle hub geometry, or mechanical stress during needle advancement. The size, weight, and structural properties of the device may also have influenced handling characteristics during the simulation task. Furthermore, the prototype was not designed for clinical use, and issues related to sterility, durability, and patient safety were not evaluated. Therefore, the present findings should not be interpreted as validation of a precision-engineered guidance device. Further refinement using lighter materials and more standardized manufacturing methods may improve mechanical stability, reproducibility, usability, and potential translational applicability, and should be evaluated in future studies.

In conclusion, this exploratory simulation-based study suggests that the needle-mounted guide may serve as a simple educational aid for novice practitioners learning freehand ultrasound-guided needle insertion, particularly in the in-plane technique. The device was associated with improved subjective needle visualizaion despite the absence of statistically significant differences in objective outcomes. These findings support its potential role as a simple adjunct in simulation-based training, although further studies are required to confirm its educational effectiveness under more standardized conditions and to evaluate its applicability to different needle-guidance techniques.

## Data Availability

The datasets used and analyzed during the current study are available from the corresponding author on reasonable request.

## Abbreviation

VAS: Visual analogue scale
